# Effect of community-level intervention on antenatal care attendance: a quasi-experimental study among postpartum women in Eastern Uganda

**DOI:** 10.1080/16549716.2022.2141312

**Published:** 2022-11-11

**Authors:** Solomon T Wafula, Aisha Nalugya, Rornald M Kananura, Richard K Mugambe, Moses Kyangwa, John B Isunju, Betty Kyobe, Tonny Ssekamatte, Sarah Namutamba, Gertrude Namazzi, Elizabeth K Ekirapa, David Musoke, Florian Walter, Peter Waiswa

**Affiliations:** a Department of Disease Control and Environmental Health, School of Public Health, College of Health Sciences, Makerere University, Kampala, Uganda; b School of Health Sciences, Faculty of Biology, Medicine and Health, The University of Manchester, Manchester, UK; c Department of Health Policy, Planning and Management, School of Public Health, College of Health Sciences, Makerere University, Kampala, Uganda; d Global Health Department of Public Health Sciences, Karolinska Institutet, Stockholm, Sweden

**Keywords:** Community health workers, antenatal care, early ANC initiation, community mobilization, Training and supervsion, Eastern Uganda

## Abstract

**Background:**

Early Initiation of antenatal care (ANC) and at least four visits during pregnancy allow screening and support for a healthy lifestyle and self-care during pregnancy however, community-directed interventions to improve access to these services are rarely explored.

**Objective:**

To assess the effect of community health worker (CHW) involvement on utilisation of antenatal services during pregnancy in resource-constrained rural settings in Uganda.

**Methods:**

We conducted a quasi-experimental evaluation study among mothers from Eastern Uganda. We used Difference in Differences (DiD) analysis to assess the effect of CHW intervention on ANC attendance. Components of the intervention included community dialogues and empowering CHWs to educate pregnant women about using maternal health services. The primary endpoints were early initiation of ANC and completion of at least 4 ANC visits.

**Results:**

Overall, the intervention significantly improved attendance of ≥ 4 ANC visits (DiD = 5.5%). The increase was significant in both intervention and comparison areas (46.2–64.4% vs. 54.1–66.8%, respectively), with slightly greater gains in the intervention area. Other elements that predicted ≥4 ANC attendance besides the intervention were post-primary education (PR1.14, 95%CI 1.02–1.30), higher wealth quintile (PR1.17, 95%CI 1.06–1.30), and early initiation of ANC (PR1.58, 95%CI 1.49–1.68). The intervention did not significantly improve early initiation of ANC (DiD =-1.3%). Instead, early initiation of ANC was associated with higher husband education (PR1.19,95%CI 1.02–1.39), larger household size (PR = 0.81, 95%CI 0.70–0.95), and higher wealth index (PR1.19,95%CI 1.03–1.37).

**Conclusions:**

The CHW intervention improved attendance of at least 4 ANC visits but not early initiation of ANC. There is need to promote CHW-led health education to increase attendance at 4+ ANC visits, but other approaches to promote early initiation are urgently required.

## Background

Maternal mortality remains a significant public health problem globally. Although substantial progress has been made in reducing maternal mortality over the last two decades, the number of maternal deaths worldwide remains unacceptably high [1]. In 2017, an estimated 295,000 women died from complications related to pregnancy and childbirth, which translates to 810 maternal deaths per day [2]. The burden of maternal mortality is disproportionately higher (94%) in low- and middle-income countries (LMICs), with Sub-Saharan Africa (SSA) accounting for two-thirds of all global maternal deaths [2]. In Uganda, the maternal mortality ratio is estimated at 336 maternal deaths per 100,000 live births [3]. Most maternal deaths result from haemorrhages, sepsis, pre-eclampsia, unsafe abortion, and congenital disabilities [4]. These complications can be prevented with appropriate maternal health services, including antenatal care (ANC) [1].

Early initiation and optimal antenatal care are essential in reducing perinatal and maternal mortality. We defined early ANC attendance as attending ANC at less than 12 weeks of gestation, while optimal ANC was defined as attending at least four visits, as defined by the World Health Organization [5]. It is essential for detecting, preventing, and treating adverse pregnancy-related outcomes [5]. In addition, it presents an opportunity to deliver evidence-based interventions that can positively influence maternal and child health. These interventions range from health education, counselling on pregnancy-related physiological changes and potential complications, vaccinations, nutritional counselling, anaemia and malaria prevention, and prevention of mother-to-child transmission of Sexually Transmitted Infections (STIs) [6].

Until recently, WHO recommended that pregnant women attend at least four ANC visits during pregnancy but start it before 12 weeks gestation [5]. However, WHO revised this recommendation in 2016 from four ANC contacts to eight, following recent evidence that eight or more ANC visits can reduce perinatal deaths by up to 8 per 1000 live births compared to 4 visits [7,8]. Achieving this recommendation will be even more difficult, especially for women in LMICs [9]. According to available literature, only 52% of the women in Sub-Saharan Africa attended at least 4 ANC visits during pregnancy [9]. In particular, long distances to facilities, lack of information, inadequate and low quality services, and cultural beliefs and practices have been identified as significant barriers to optimal ANC attendance [2]. Given these barriers, it has been suggested that community-based interventions can improve the access and uptake of maternal health services [10–12].

Community-level interventions may involve community support groups/women groups, community mobilisation, community dialogues, home visits and training of community health workers (CHWs) [10]. Existing evidence suggests that such interventions can improve breastfeeding practices and increase referrals to healthcare facilities for pregnancy-related complications and other healthcare services during pregnancy [13]. In northern Uganda, an Intervention involving the CHW component to track women who had missed ANC appointments through Village Health Team (VHT) training, home visits, and community dialogues, significantly increased ANC attendance and skilled birth attendance [14].

Whereas community-based interventions are essential in improving maternal and neonatal health [13], there is limited research on using community health workers to promote early initiation and attendance of ≥ 4 ANC visits. A two-year maternal and neonatal project was implemented in eastern Uganda from 2011 to 2013 to provide policymakers with evidence for scalable maternal and child care packages in rural Ugandan settings. The intervention included 3–5 community health workers (CHWs) per village visiting homes (at least two during pregnancy) to provide health education to mothers before and during pregnancy. These CHWs also held community dialogues (quarterly) to promote antenatal care attendance, skilled birth delivery, and prevention of mother-to-child transmission (PMTCT). Prior to deployment, these CHWs underwent five days of intensive training, after which district and national level instructors supervised their work. We maintained the standard approach in the control areas, and CHWs continued to work without project-related training, supervision or facilitation. The project team did not inform them about implementing any interventions in other districts. A pre-intervention household-based cross-sectional survey was conducted in the intervention and control HSDs to determine the baseline uptake of ANC, early ANC initiation, and attendance of ≥ 4 ANC visits during the previous pregnancy. After that, the community-based intervention was only implemented in the intervention district for three years before an endline evaluation was conducted in both areas. The overall objective was to assess the effect of this community-led intervention on early initiation of ANC and attendance of ≥ 4 ANC visits in intervention and control districts, as well as the factors associated with these ANC attendance practices among women in Eastern Uganda.

## Methods

### Study design and setting

This household study was conducted in three districts in the South-Eastern part of Uganda (Busoga region). Buyende and Luuka were among the intervention districts, whereas Iganga served as the control district. Busoga region has about 3 million people, or 10% of Uganda’s population, covering an area of about 7100 sq. miles [3]. The region has ten administrative districts. Iganga District is also home to the Iganga/Mayuge Health Demographic Surveillance Site (HDSS), thus providing an opportunity for ongoing tracking of coverage, equity, and trends of key indicators. The HDSS area is predominantly rural but partly peri-urban in some trading areas. The three districts where the study was conducted have an estimated population of 1,100,000 people [15] and have similar rates of maternal and health services utilisation indicators.

This study employed a quasi-experimental, non-equivalent group, pre-test–post-test design. We chose a quasi-experimental design since RCT would be impractical due to the group nature of the intervention. In contrast to RCTs, which take place in highly controlled settings, quasi-experimental design also offers practical options for conducting impact evaluations in real-world settings. The results of this design may be highly relevant for guiding policy. We conducted two cross-sectional surveys (baseline and follow up). We assessed the same outcomes at baseline and endline.

### Study population, units and eligibility criteria

The study units were households. Women who were pregnant within the previous year and lived in the study area, regardless of whether the baby was born preterm or full-term, and irrespective of the birth outcome, made up the study population (whether the baby was alive or dead). Women with severe illness or who had not lived in the community for at least one year were excluded.

### Sample size and sampling procedures

A baseline sample of 1,582 postpartum women in the intervention area and 388 women from the control areas were interviewed. During the endline evaluation, we interviewed a similar sample size of 1,661 and 401 mothers from intervention areas and control areas. Regarding sampling, 16 sub-counties were randomly selected (6 in each intervention district and 4 in the control district) by writing the names of all sub-counties on small pieces of paper, then picking one paper at a time without replacement until all sub-counties were obtained. We randomly selected one parish within each sub-county, and two villages were chosen randomly from each parish. With the help of local leaders, we compiled a list of all mothers who had given birth within the previous 12 months of the surveys. The respondents were sampled at the village level using simple random sampling from a village listing created with the assistance of the local council 1 (village) leader. Enumerators visited at least 50 households in the selected/sampled village, selecting one eligible respondent per eligible household. The same villages were considered in the 2015 endline evaluation. A similar approach was used to determine a cross-sectional sample of mothers who gave birth in 2014 for the endline survey.

### Theory of change

Complex interventions, such as the CHW program, require a convincing theory of change. This theory should be supported by a well-described hypothesis of intervention-to-outcome pathways [16]. The theory of change adopted for this study has been described in an earlier publication [11]. In brief, we expected the home visits made by CHWs and community dialogues to empower pregnant women to encourage ANC uptake, particularly through early initiation and completion of at least four ANC visits at the healthcare facility. CHWs would accomplish this through activities such as (i) counselling, which raises awareness of ANC and can serve as a source of motivation, as previously demonstrated [17]; (ii) informing pregnant women of the location of the nearest healthcare facility that provides ANC; (iii) the visit itself serving as a reminder or ‘nudge’ to women who were already planning to attend ANC or deliver at a healthcare facility; and (iv) CHWs exerting a normative social influence on women through their visits and by emphasising the importance of ANC and other maternal health services. We hypothesised that these activities would lead to an improvement in the timeliness and frequency of ANC uptake when compared with standard care.

### Data collection

Appointments were made with CHWs and local village council (LC) leaders of the selected villages before the data collection exercise. One CHW and a representative from the LC led the Research Assistants (RAs) to the households that had been chosen. Data were collected from the mothers in selected homes through face-to-face interviews using a structured questionnaire developed based on reviewed literature on antenatal care attendance and community-based approaches [18–21]. The same questionnaire was used for both the baseline and follow-up studies. Since the baseline and endline were independent cross-sectional surveys, participants in the post-intervention survey included both new and baseline survey participants. Data on sociodemographic characteristics (age, parity, household size, highest education level, wealth index, occupation, and marital status) and maternal and child health service utilisation (ANC attendance and health facility deliveries) were collected. The questionnaires were filled out by experienced research assistants (RAs) who were well trained in objective interviewing. After completing the interviews, each RA compiled their completed questionnaires and turned them over to supervisors, who reviewed the tools, checked for errors, and completed the compilation.

### Variables

- The key outcome variables were Early ANC initiation and attendance of ≥ 4 ANC visits. Early ANC initiation was defined as making first contact (first visit) with a health worker within the first trimester (≤12 weeks/or ≤3 months) of gestation. ANC attendance was self-reported and confirmed by checking the ANC attendance card. The first visit within 12 weeks of conception was coded “1” and otherwise coded “0”. Having four or more ANC visits was coded “1” and otherwise coded “0”.
- The primary exposure was the Intervention (CHW home visits and community dialogue): This was a group variable, and mothers were considered to have received the intervention if they resided in the intervention district; otherwise, not.
- The confounders and covariates for this association included sociodemographic and individual characteristics of women such as mother’s age, gender, marital status, educational level of women and husbands, parity, occupation and socioeconomic status (SES). The level of education was recorded as none, primary, or post-primary. Marital status was divided into two categories: married/living with a partner and unmarried. Occupations were classified as “peasant farmer”, “housewife”, “business and other occupations”, while parity was categorised into “primiparous”, “2 – 4”, and “5 and more”. SES (as determined by wealth asset index): The wealth quintiles were created using principal components analysis (PCA) based on data on assets and household structure using the Uganda Bureau of Statistics (UBOS) criteria [3].

### Quality control

Before the baseline and endline surveys, research assistants were trained. The questionnaire and consent forms were available both in English and the local language (*Lusoga*), with the latter being the primary language used in data collection. Tools were pre-tested in five villages of Namungalwe sub-county in order to fine-tune the questionnaires to meet the required objectives set for evaluation. These villages had similar characteristics to study area settings but were not included in the final data collection. Questionnaires containing information from every participant in the study were kept securely, and only the study team had access to them. Data entry was performed by independent, experienced data entry clerks who had received two days of training. We kept all files on password-protected computers.

### Statistical analyses

Continuous variables such as age were summarised as mean and standard deviation and compared between baseline and endline using two-sample *t*-tests. Categorical data, on the other hand, were expressed as frequencies and proportions and compared between baseline and endline using Pearson’s chi-squared tests. To explore the contribution of the intervention package to early initiation of ANC and completion of at least four ANC visits, a modified Poisson regression incorporating the difference in differences (DiD) analyses (Equation (1)) with a less strict exchangeability assumption was used. The data were entered into Epi info 7 and then transferred for analysis into Stata 16.0 (StataCorp, College Station, Texas, USA).

$y_{it}=\beta_{0}$+$\beta_{1}P_{T}$+$\beta_{2}T_{i}$ +$\beta_{3}$($P_{t}$*$T_{i}$) +$\mu_{it}$ (1)

where yit is the outcome of interest, P is a dummy variable during the time period ‘t’, and T is a dummy variable for the treatment group. The interaction term, P × T, is equivalent to a dummy variable equal to 1 for observations in the treatment group in the second period. β3 is the DiD estimator, indicating whether the expected mean change in outcomes before and after the intervention differed between the intervention and control groups. We ran separate models for each study outcome. Multivariable modified Poisson regression was performed to understand the predictors of the study outcomes (early ANC initiation, optimal ANC attendance) after adjusting for known confounders such as age and level of education. Variables with *p* values ≤0.25 at the univariate level were known confounders, and variables we judged biologically plausible were considered for multivariate analysis. Hosmer – Lemeshow was used to assess the model’s goodness of fit [22].

### Ethical considerations

We obtained formal approval from Makerere University School of Public Health Higher Degrees, Research and Ethics Committee (HDREC), the Uganda National Council for Science and Technology (UNCST) and the WHO Ethical Review Committee. Additionally, the study was approved by the district health teams and by the local authorities where the study was conducted. Before participating in the study, participants (adults and emancipated minors) provided written informed consent. The research assistants read an informed consent document to participants (in either English or the local language). All research procedures were conducted following the principles of the Helsinki Declaration, and all the activities followed standard operating procedures and codes of conduct.

## Results

### Sociodemographic characteristics of participants of participants

The study included 4,032 women (1970 at baseline and 2,062 at endline evaluation). At baseline, the mothers’ ages ranged from 13 to 53 years, with an average age of 26.2 (SD = 6.6). Majority of the women were peasant farmers (81.3%) and had primary as their highest level of education (67.3%). At baseline, the distributions of women’s age, parity, and level of education were similar across the intervention and control arms. Approximately 26.9% and 35.7% of women in the control and intervention groups had initiated ANC early (*p = 0.001*). In addition, 54.1% and 47.8% of the women in control and intervention arms had completed four ANC visits (*p = 0.006*) (Table 1).

**Table 1.** Distribution of sociodemographic characteristics by study arm at baseline and endline.

	Baseline (N = 1970)			Endline (N = 2062)		
Overall	Control, n (%) 388 (100)	Intervention, n (%) 1582(100)	p-value	Control, n (%) 401 (100)	Intervention, n (%) 1661 (100)	p-value
**Age group (in years)**						
<20	71 (19.3)	251 (16.8)	*0.416*^*a*^	44 (11.4)	186 (11.9)	*0.815*^*a*^
20 – 24	96 (26.1)	413 (27.6)		117 (30.2)	511 (32.7)	
25 – 29	89 (24.2)	388 (25.9)		91 (23.5)	368 (23.5)	
30 – 34	54 (14.7)	249 (16.6)		73 (18.9)	265 (17.0)	
≥35	58 (15.8)	196 (13.1)		62 (16.0)	233 (14.9)	
Mean Age ± SD	26.3 ± 7.0	26.3 ± 6.6	*0.845*^b^	27 ± 6.6	26.7 ± 6.6	*0.320*^b^
**Highest education level**						
None	57 (14.7)	191 (12.1)	*0.245*^*a*^	45 (11.2)	201 (12.1)	*0.181*^*a*^
Primary	248 (63.9)	1075 (68.0)		239 (59.6)	1046 (63.2)	
Post-primary	83 (21.4)	314 (19.9)		117 (29.2)	409 (24.7)	
**Husband’s highest level of education**						
None	23 (6.7)	132 (9.3)	*0.087*^*a*^	53 (14.3)	205 (13.2)	***0.027***^*a*^
Primary	173 (50.7)	770 (54.0)		161 (43.3)	791 (50.9)	
Post-primary	145 (42.5)	525 (36.8)		158 (42.5)	558 (35.9)	
**Parity**						
Primiparous	72 (18.6)	255 (16.1)	*0.515*^*a*^	72 (18.0)	290 (17.5)	*0.949*^*a*^
2 – 4	154 (39.7)	648 (41.0)		176 (43.9)	740 (44.6)	
5 and more	162 (41.8)	578 (42.9)		153 (38.2)	623 (37.8)	
**Marital status**						
Married	350 (90.2)	1440 (91.2)	*0.541*^*a*^	364 (90.8)	1530 (92.3)	*0.319*^*a*^
Not married	38 (9.8)	139 (8.8)		37 (9.2)	128 (7.7)	
**Occupation**						
Peasant farmer	292 (75.3)	1308 (82.9)	***<0.001***^*a*^	199 (49.6)	1019 (61.5)	***<0.001***^*a*^
Housewife	50 (12.9)	114 (7.2)		149 (37.2)	405 (24.5)	
Business and other occupation	46 (11.9)	156 (9.9)		53 (13.2)	232 (14.0)	
**Timing of first ANC visit**						
≤12 **weeks**	103 (26.9)	552 (35.7)	***0.001***^*a*^	161 (40.7)	790 (48.1)	***0.007***^*a*^
>12 **weeks**	280 (73.1)	994 (64.3)		235 (59.3)	851 (51.9)	
**Number of ANC visits**						
1 – 3	174 (45.9)	828 (53.8)	***0.006***^*a*^	130 (33.3)	580 (35.6)	*0.376*^*a*^
4+	205 (54.1)	712 (47.8)		261 (66.7)	1048 (64.4)	

ANC: Antenatal Care, SD: Standard Deviation, ^*a*^ *P Values* based on chi-squared statistics, ^b^ *P Values* based on two sample (independent) *t*-test.

**Figure 1.**
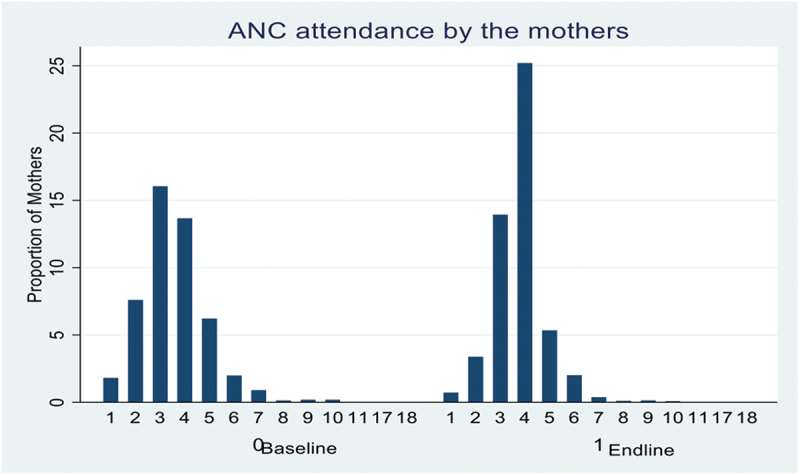
Number of ANC visits made at baseline and end line.

At the endline, the average age of mothers was 26.8 (SD = 6.6). More than half were peasants (59.2%) and had primary as their highest level of education (62.5%). Differences in occupation (p < 0.001) and early ANC initiation (p = 0.007) persisted at the endline, while differences in the number of ANC visits were no longer statistically significant. Differences in the husband’s level of education, which were not statistically significant at the baseline, were statistically significant at the endline (p = 0.027) (Table 1). Figure 1 shows the overall frequency of ANC visits before and after the intervention.

### Effect of the intervention on early ANC timing and making of at least 4 ANC visits

At baseline, 35.7% of women in the intervention area had early ANC attendance during their last pregnancy, which increased to 48.1% (p < 0.001) at the endline. In the control area, 26.9% of the women presented early for ANC at baseline, and this proportion increased to 40.7% (p < 0.001) at the endline. The net change of −1.3% (DiD, *p* = 0.502) in the intervention area compared to changes in the control area was due to an increase of 12.4% in the intervention area and 13.8% in the control area.

Regarding attendance of ANC at least four times, 46.2% of women in the intervention area had at least four ANC visits at baseline, which significantly increased to 64.4% (+18.2) (p 0.001) at the endline. Similarly, in the control area, 54.1% of the women had at least four ANC visits which increased to 66.8% (+12.7) (p < 0.001). However, the net improvement (DiD = +5.5, *p* = 0.037) in the intervention area over the control area was significant (Table 2).

**Table 2.** Effect of intervention on Early imitation of ANC and attendance of at least 4 ANC visits.

Indicators	Baseline (*n* = 1970)			Endline (*n* = 2062)			DiD	*p values*
	C (%)	I (%)	Diff (1-C) (%)	C (%)	I (%)	Diff (1-C) (%)		
ANC attendance during pregnancy	99.2	98.2	− 1.1(*p* = 0.078)	99.3	99.3	0.1(*p* = 0.884)	+1.2	0.174
Early ANC initiation	26.9	35.7	+8.8(*p* = 0.001)	40.7	48.1	+7.5(*p* = 0.006)	− 1.3	0.502
At least 4 ANC visits during pregnancy	54.1	46.2	− 7.9(*p* = 0.005)	66.8	64.4	− 2.4(*p* = 0.387)	+5.5	0.037

C: control area; I: intervention area; Diff: difference; DiD: difference in differences; ANC: Antenatal Care.

### Factors associated with early initiation of antenatal care

In the multivariable modified Poisson regression analysis, women whose husbands had post-primary education had a 19% higher likelihood of attending ANC in their first trimester than those whose husbands had no formal education (PR = 1.19, 95%CI 1.02–1.39). Similarly, the adjusted analysis revealed that women in the second (PR = 1.18, 95%CI 1.04–1.35), third (adjusted PR = 1.19, 95%CI 1.04–1.36), and fifth wealth quintiles (PR = 1.19, 95%CI 1.03–1.37), were 18%, 19% and 19% respectively more likely to initiate ANC early in the first trimester than women in the first wealth quintile. Women from families with at least 8 members were 19% less likely to start ANC early in their first trimester than those with 4 or fewer members (PR = 0.81, 95%CI 0.70–0.95) (Table 3).

**Table 3.** Factors associated with early ANC initiation.

	Early ANC initiation		Crude	*p-value*	Adjusted	*p-value*
	No	Yes	PR (95% CI)		PR (95% CI)	
**Age group (in years)**						
<20	331 (60.6)	215 (39.4)	1		1	
20 – 24	603 (53.8)	518 (46.2)	1.17 (1.04–1.32)	0.010*	1.15 (0.99–1.34)	0.061
25 – 29	543 (59.1)	376 (40.9)	1.04 (0.91–1.18)	0.563	1.02 (0.86–1.22)	0.776
30 – 34	385 (60.8)	248 (39.2)	0.99 (0.86–1.15)	0.944	1.02 (0.83–1.25)	0.828
≥35	369 (68.6)	169 (31.4)	0.80 (0.68–0.94)	0.006**	0.88 (0.70–1.11)	0.270
**Highest education level**						
None	309 (64.5)	170 (35.5)	1		1	
Primary	1555 (60.4)	1016 (39.5)	1.11 (0.98–1.27)	0.105	1.02 (0.88–1.19)	0.778
Post-primary	491 (54.0)	418 (46.0)	1.30 (1.13–1.49)	<0.001***	1.08 (0.91–1.28]	0.376
**Husband’s highest level of education**						
None	250 (61.7)	155 (38.3)	1		1	
Primary	1154 (61.9)	711 (38.1)	1.00 (0.87–1.14)	0.956	1.05 (0.90–1.22)	0.533
Post-primary	748 (54.6)	622 (45.4)	1.18 (1.03–1.36)	0.014*	1.19 (1.02–1.39)	0.025*
**Parity**						
Primiparous	380 (56.0)	299 (44.0)	1		1	
2 – 4	952 (56.3)	739 (43.7)	0.99 (0.89–1.10)	0.882	0.97 (0.85–1.11)	0.678
5 and more	1020 (64.3)	567 (35.7)	0.81 [0.73–0.90]	<0.001***	0.98 (0.82–1.17)	0.817
**Marital status**						
Married	2151 (59.3)	1477 (40.7)	1			
Not married	205 (61.8)	127 (38.3)	0.94 (0.82–1.08)	0.391		
**Occupation**						
Peasant farmer	1708 (61.7)	1060 (38.3)	1		1	
Housewife	388 (54.8)	320 (45.2)	1.18 (1.07–1.30)	0.001**	0.99 (0.89–1.10)	0.877
Business and other occupation§	258 (53.6)	223 (46.4)	1.21 (1.09–1.35)	<0.001***	1.03 (0.91–1.18)	0.625
**Household size**						
1 – 4	682 (54.4)	572 (45.6)	1		1	
5 – 8	1136 (59.8)	763 (40.2)	0.88 (0.82–0.96)	0.002**	0.94 (0.89–1.10)	0.218
≥8	537 (66.5)	270 (33.5)	0.73(0.65–0.82)	<0.001**	0.81 (0.70–0.95)	0.010*
**Wealth index**						
Lowest	469 (63.0)	275 (37.0)	1		1	
Second	456 (58.8)	319 (41.2)	1.11 (0.98–1.26)	0.094	1.18 (1.04–1.35)	0.013*
Middle	445 (57.2)	333 (42.8)	1.16 (1.03–1.31)	0.021*	1.19 (1.04–1.36)	0.011*
Fourth	452 (63.1)	264 (36.9)	1.00 (0.87–1.14)	0.971	1.04 (0.90–1.20)	0.641
Highest	404 (55.2)	328 (44.8)	1.21 (1.07–1.37)	0.002*	1.19 (1.03–1.37)	0.020*
**Period**						
Baseline	1274 (66.0)	655 (34.0)	1		1	
Endline	1086 (53.3)	951 (46.7)	1.37 (1.27–1.49)	<0.001***	1.63 (1.29–2.07)	<0.001***
**Received Intervention**						
No	515 (66.1)	264 (33.9)	1		1	
Yes	1845 (57.9)	1342 (42.1)	1.24 (1.12–1.38)	<0.001***	1.41 (1.15–1.74)	**0.001**
Period* intervention (DID)					0.84 (0.66–1.09)	0.196

**CI**: confidence interval**; PR =** Prevalence ratio; Inference *** p < 0.001; ** p < 0.01; *p < 0.01, +p <0.1.

### Factors associated with optimal ANC attendance (at least 4 ANC visits)

In the multivariable modified Poisson regression analysis, women who began their first ANC in their first trimester (early initiation) were at least 1.5 times more likely to attend ANC four times or more than those who did not start ANC early. (PR = 1.58, 95%CI 1.49–1.68). We also found that women with post-primary education were 14% more likely to attend ANC four times or more than those without formal education (PR = 1.14, 95%CI 1.02–1.30). Women in the highest (fifth) wealth quintile were 17% more likely to attend ANC at least four times than those in the lowest (first) quintile (PR = 1.17, 95%CI 1.06–1.30).

The estimated effect of the intervention (DID) shows that the intervention increased the probability of attending ANC at least 4 times by 16% (adjusted PR = 1.16, 95%CI 1.01–1.33) (Table 4).

**Table 4.** Factors associated with optimal ANC attendance (4+ Visits).

Variables	At least 4 ANC visits		Crude		Adjusted	
	No	Yes	PR [95% CI]	*p-value*	PR [95% CI)	*p-value*
**Age group (in years)**						
<20	218 (40.4)	322 (59.6)	1		1	
20 – 24	457 (40.8)	662 (59.2)	0.99 (0.91–1.08)	0.855	0.95 (0.86–1.05)	0.311
25 – 29	392 (42.8)	523 (57.2)	0.95 (0.88–1.05)	0.353	0.95 (0.84–1.07)	0.388
30 – 34	285 (45.3)	344 (54.7)	0.92 (0.83–1.01)	0.088+	0.97 (0.84–1.11)	0.624
≥35	249 (47.0)	281 (53.0)	0.89 (0.80–0.99)	0.030*	0.98 (0.84–1.13)	0.760
**Highest education level**						
None	249 (52.5)	225 (47.5)	1		1	
Primary	1151 (45.1)	1403 (54.9)	1.16 (1.05–1.28)	0.005**	1.08 (0.96–1.20)	0.206
Post-primary	311 (34.4)	593 (65.6)	1.38 (1.24–1.54)	< 0.001***	1.14 (1.02–1.30)	0.028*
**Husbands’ highest level of education**						
None	201 (50.6)	196 (49.4)	1		1	
Primary	852 (45.9)	1003 (54.1)	1.10 (0.98–1.22)	0.099+	1.05 (0.94–1.16)	0.407
Post-primary	506 (37.2)	855 (62.8)	1.27 (1.14–1.42)	< 0.001***	1.09 (0.98–1.22)	0.119
**Parity**						
Primiparous	244 (36.2)	431 (63.9)	1		1	
2 – 4	711 (42.2)	979 (57.8)	0.91 (0.84–0.97)	0.005**	0.94 (0.86–1.03)	0.215
5 and more	754 (48.1)	815 (51.9)	0.81 (0.76–0.88)	< 0.001***	0.93 (0.82–1.05)	0.237
**Marital status**						
Married	1558 (43.3)	2043 (56.7)	1			
Not married	152 (45.8)	180 (54.2)	0.96 (0.86–1.06)	0.387		
**Occupation**						
Peasant farmer	1294 (47.0)	1456 (53.0)	1		1	
Housewife	256 (36.4)	448 (63.6)	1.20 (1.13–1.29)	< 0.001***	1.02 (0.95–1.09)	0.647
Business and other occupation§	160 (33.6)	316 (66.4)	1.25 (1.17–1.35)	< 0.001***	1.05 (0.96–1.15)	0.263
**Household size**						
1 – 4	479 (38.4)	767 (61.6)	1		1	
5 – 8	838 (44.4)	1049 (55.7)	0.90 (0.85–0.96)	0.001**	0.99 (0.91–1.06)	0.676
≥8	393 (49.1)	407 (50.9)	0.83 (0.76–0.90)	< 0.001***	0.96 ([0.86–1.06)	0.376
**Wealth index**						
Lowest	379 (50.8)	367 (49.2)	1		1	
Second	326 (42.7)	437 (57.3)	1.16 (1.05–1.28)	0.002**	1.09 (0.99–1.20)	0.068+
Middle	328 (42.3)	447 (57.7)	1.17 (1.07–1.29)	0.001**	1.09 (0.99–1.20)	0.078+
Fourth	323 (45.6)	385 (54.4)	1.11 (1.00–1.22)	0.048*	1.04 (0.94–1.16)	0.400
Highest	250 (34.5)	474 (65.5)	1.33 (1.22–1.46)	< 0.001**	1.17 (1.06–1.30)	0.001**
**Early ANC initiation**						
No (> 12 weeks)	1308 (55.9)	1034 (44.2)	1		1	
Yes (≤ 12 weeks)	393 (24.9)	1185 (75.1)	1.70 (1.62–1.79)	< 0.001***	1.58 (1.49–1.68)	< 0.001***
**Period**						
Baseline	1002 (52.2)	917 (47.8)	1		1	
Endline	710 (35.2)	1309 (64.8)	1.36 (1.28–1.44)	< 0.001***	1.13 (1.00–1.27)	0.055+
**Received Intervention**						
No	304 (39.5)	466 (60.5)	1		1	
Yes	1408 (44.4)	1760 (55.6)	0.92 (0.86–0.98)	0.010*	0.82 (0.73–0.92)	0.001**
Time* intervention (DiD)					1.16 (1.01–1.33)	0.036*

**CI**: confidence interval**; PR =** Prevalence ratio; Inference *** *p*<0.001; ** *p*<0.01; *p<0.01, +p<0.1.

## Discussion

This study sought to assess the effect of a community-level intervention on early and optimal (at least 4 visits) ANC attendance and the factors associated with ANC attendance practices.

### Effect of CHW intervention on antenatal care

These findings demonstrate the potential power of CHWs to effect change in the uptake of antenatal care practices, which is consistent with previous research [23]. Although the intervention did not significantly improve early ANC initiation, which has its inherent benefits, it substantially improved attendance at 4 ANC visits. Increased ANC attendance may offer opportunities to spot complications and reduce their severity, potentially lowering the risk of maternal and infant mortality. However, a lower proportion of women attained ≥4 visits than recommended by the national guidelines, raising concerns about the viability of the current WHO recommendation for at least 8 ANC visits during pregnancy. We believe that the observed changes in attendance at 4 ANC visits are primarily due to the CHW intervention due to confounder adjustment in the DID analysis. Although estimates remain lower than recommended by WHO guidelines, there was an overall increase in early ANC initiation rates and attendance at 4 ANC visits in both intervention and control arms. The general improvement in ANC attendance, including early ANC initiation across both intervention and control arms, may be explained at least in part by the secular trend towards better maternal and Newborn care. Although it is evident that CHWs exist in all districts and identify and visit pregnant women, as well as educate and refer them for hospital care, including ANC, the higher net increase is due to additional incentives and supervision from the project team in the intervention area. The CHW strategy was nationally popularised, and the Maternal and Perinatal Death Review Strategy was implemented in all districts [24]. Moreover, a similar intervention had been implemented in the control area (Iganga) just one year before this study which may partly explain the non-significant effect of this intervention [25].

Early initiation of antenatal care allows for early detection and management of complications [26]. Attendance of at least 4 ANC visits was significantly associated with receiving the community intervention. CHWs’ efforts, such as sensitising mothers about the importance of timely uptake of ANC and at least 4 ANC visits through routine home visits, could have convinced mothers to attend ANC early. Similarly, recent intervention studies have shown that trained and supervised CHWs can successfully identify and visit pregnant women at home to promote various healthy practices, including early and optimal ANC attendance [25,27]. In India, a similar study found that CHWs can encourage mothers to consider early ANC initiation and retention in the maternity care continuum [28]. As a result, CHW interventions are essential in promoting maternal health and have significant policy and practice implications in Uganda and other similar settings where CHW programmes for maternal and Newborn care are being designed or scaled up.

### Factors associated with early initiation of ANC attendance

Other than the intervention, which showed no effect on early ANC initiation, other findings indicated that increasing household wealth status significantly impacted early ANC initiation among the women. Women in higher quintiles of wealth were more likely to go to ANC in the first trimester than those in the lowest quintile. Our results are consistent with past findings in cross-sectional studies from Ghana, Nigeria and Nepal in which a correlation between an increase in wealth status and ANC attendance was highlighted [29–31]. Compared to their counterparts, women in higher wealth groups can afford the costs associated with accessing maternal healthcare, such as transportation costs to and from the healthcare facility and health services. Indeed, previous studies have shown that the cost of health services is a significant factor in determining late ANC attendance. As a result, the cost of accessing care is likely to be a barrier to the timely initiation of ANC among women from lower-income households [32]. Therefore, with a high proportion of mothers in the Busoga region living in poverty, interventions such as savings, transport vouchers and community insurance programs that focus on the economic empowerment of women and their families could promote early initiation of ANC.

Early women’s initiation of ANC was associated with higher education of the husband. In this study, first-trimester attendance at ANC was 19% higher for women whose husbands had post-primary education than those without formal education. Similarly, earlier research found that paternal education level was a statistically significant predictor for early ANC initiation [33,34]. We believe husbands with higher levels of education are more likely to be aware of issues relating to maternal healthcare, including ANC attendance, and to have greater financial access to these services. As a result, they may be able to influence and encourage their women to start the ANC early. Additionally, research shows that men’s roles as family patriarchs and primary decision-makers significantly impact women’s decisions about their health and behaviour [35]. Therefore, men’s education level is vital in improving maternal and neonatal health decision-making.

### Factors associated with ANC attendance of 4 or more visits

Early initiation for ANC was strongly associated with attendance of ≥ 4 ANC visits. In our study, women who started their first ANC in the first trimester were 1.5 times more likely to attend ANC at least four times than those who did not begin their first ANC in the first trimester. Previous studies on the determinants of ANC visits showed that late initiation of ANC was negatively associated with attendance of 4 or more ANC visits [27,36]. Early initiation of ANC is a crucial opportunity for providing medical care, health education, and essential information on the status of their pregnancy [37], which in turn informs ongoing ANC attendance throughout pregnancy. This finding suggests that timely initiation of ANC during pregnancy leads to optimal ANC attendance. Strategies that promote early ANC attendance should be emphasised.

The study’s findings also showed that women with post-primary education were 14% more likely to attend at least ANC visits than women with no formal education. These results corroborate those in studies conducted in Tanzania and Nigeria, where higher maternal education was significantly associated with optimal attendance of ANC [38,]. This may be because increased education levels among women are frequently accompanied by employment, financial security, and a greater understanding of the advantages of health services, leading to better access to maternal health care. Moreover, women with higher education are more likely to recognise the beneﬁts of using maternal healthcare services [39]. Women’s confidence and autonomy in making decisions within the home are both boosted by education [39]. Therefore, educated women are more likely to make better and wise decisions about their health. Therefore, more education about the advantages of ANC is required, especially for women with low levels of education.

Women in the highest (fifth) wealth quintile had a 17% higher likelihood of attending ANC at least four times than those in the lowest (first) wealth quintile. A higher income level ensures economic and physical access to maternal healthcare services. In particular, where there are significant travel distances to healthcare facilities, as in these rural settings, the cost of accessing care (travel costs, service fees, equipment costs) is a key factor in determining whether or not to use MCH services [40,41]. As a result, women in lower wealth quintiles are less likely to attend ANC regularly and, therefore less likely to meet the recommended optimal guidelines. Our findings support previous studies that low incomes or wealth indices were associated with predicted low ANC utilisation rates [38,42, 43]. As was previously mentioned, interventions geared toward the economic empowerment of women and their families may increase the number of ANC visits.

## Strengths and limitations of the study

The study involved a large sample size and several districts; hence the results may be generalised to the eastern region of Uganda and other similar settings. Despite the above strengths, there were some limitations to the study, so readers should interpret the findings cautiously. Some of this study’s findings might have been impacted by recall bias because some respondents hadn’t given birth in almost a year and might not have remembered every detail of their pregnancies. However, the authors’ assumption and belief are that events surrounding pregnancy and childbirth are significant events and that mothers often have memories of them. Second, the control area (Iganga) had recently undergone a similar intervention, which may help to partially explain why the CHW intervention had no statistically significant impact on early initiation. Thirdly, we had a relatively small control group compared to the intervention group (with a ratio of approximately 1:4) due to limited funding and this was because initially, this was planned as a pre-post comparison. Later, we included the control component for more reliable effect estimates; nevertheless, the control group still had sufficient numbers (n > 400 participants) to allow for reasonable comparisons. The fourth limitation is that outcomes were partly self-reported, which may have introduced social desirability bias. However, we minimised this by highlighting the significance of the study findings and assuring participants that their responses would be kept private and confidential. Lastly, a quasi-experimental design has its inherent limitations. For example, it might not have addressed all confounding which is often best addressed in a randomised clinical trial. Furthermore, we anticipated high interventional fidelity (that all participants in the intervention area would receive the intervention), which is not always the case. However, with frequent visits and supervision, we are confident that a large proportion would have received the intervention on several occasions.

## Conclusions

The community intervention showed the potential to significantly increase the uptake of ≥ 4 ANC visits in the intervention group over the control group, reinforcing the need to scale up interventions using supported and incentivised CHWs. However, the improvement in ANC uptake was lower than the Ministry of Health guidelines, raising concerns about whether or not the 8 ANC visits that were recently advised by WHO would be feasible. In conclusion, CHWs play an essential role in delivering MCH services in rural settings, but they also require support, financial incentives and supervision to perform effectively.

## Data Availability

The data set used and analysed in this study are available from the first author upon reasonable request.

## Paper context

The prevalence of early ANC initiation and having at least 4 ANC visits throughout pregnancy is low in Uganda. Attendance of at least 4 ANC visits during pregnancy increased due to training community health workers to regularly provide health education, dialogues, and home visits to pregnant women in their communities. As CHWs may have a significant impact on the use of life-saving maternal and child health services, governments should make substantial efforts to incentivise and promote their work.
